# Lipid-capped transistors bring cardiac testing closer to the bedside

**DOI:** 10.1093/nsr/nwag403

**Published:** 2026-07-01

**Authors:** Cong Lin, Xiaohui Wang

**Affiliations:** Interdisciplinary Laboratory for Frontier Chemistry, Changchun Institute of Applied Chemistry, Chinese Academy of Sciences, China; Interdisciplinary Laboratory for Frontier Chemistry, Changchun Institute of Applied Chemistry, Chinese Academy of Sciences, China

Cardiovascular disease (CVD) remains a leading cause of global mortality, and acute myocardial infarction (AMI) is among the most time-sensitive clinical emergencies: the earlier myocardial injury is detected, the greater the opportunity for timely intervention [1–3]. Conventional laboratory assays, although accurate, usually depend on centralized instruments, trained personnel and serial sampling. Point-of-care testing (POCT) promises faster clinical decisions in emergency rooms, ambulances and primary-care settings [4,5], but electrical biosensors face a persistent obstacle in real biological fluids: high ionic strength screens target charges, while abundant serum proteins cause non-specific adsorption and false signals [6,7].

In a recent paper in *National Science Review*, Yuan *et al.* report a High-performance Electrical Assay for CVD Testing, termed HEArT, that addresses this long-standing interface problem through a biomimetic lipid-capped field-effect transistor (FET) design [8]. Crucially, the central innovation of this work lies not merely in achieving multi-biomarker cardiovascular testing, but rather in the rational construction of a lipid-capped FET interface that enables effective electrical signal transduction directly in high-ionic-strength blood-derived matrices such as serum or plasma while simultaneously suppressing non-specific protein adsorption. The platform integrates a disposable multi-channel biosensing array, a portable reader and a smartphone application. Its core component is a monolayer phospholipid interface-capped FET (MPI-FET), in which antibodies are positioned close to the indium gallium zinc oxide transistor surface, while a self-assembled dipalmitoyl phosphatidylcholine layer functions as a dual-functional sensory interface [8].

This interface is the key conceptual advance. First, the phospholipid monolayer creates an ion-regulating interfacial layer that displaces mobile ions away from the transistor surface and extends the effective electrical sensing range, thereby mitigating Debye screening and enabling antigen–antibody binding events to be transduced in serum [8]. Second, the zwitterionic headgroups generate a dense hydration layer that resists non-specific protein fouling, preserving specificity in complex biological matrices [8]. Thus, the key advance is not simply that the assay is more sensitive, but that it makes electrical sensing genuinely compatible with clinical serum samples.

Leveraging this interface, HEArT uses a 10-transistor array to simultaneously quantify 5 myocardial injury biomarkers: cardiac troponin I (cTnI), myoglobin (Myo), creatine kinase isoenzyme (CK-MB), N-terminal pro-brain natriuretic peptide (pro-BNP) and D-dimer [8]. The assay reaches low-pg/mL detection limits, spans a dynamic range of ∼5 orders of magnitude and maintains quantitative accuracy comparable to enzyme-linked immunosorbent assay (ELISA), a standard biochemical method widely used in cardiac biomarker testing [8]. In beagle AMI models, HEArT detected significant biomarker elevations within 15–45 min after myocardial injury, >30 min faster than conventional biochemical assays [8]. In 265 clinical serum samples, linear discriminant analysis condensed the five-biomarker profile into an interpretable myocardial injury signature, achieving 91.7% accuracy for AMI identification and 79.6% overall accuracy for CVD classification [8]. The platform was also applied to longitudinal monitoring after percutaneous coronary intervention, suggesting potential value for detecting myocardial ischemia–reperfusion injury risk.

The real novelty of this work lies in closing the loop from ‘interface engineering to sensor arrays, multi-biomarker algorithms and mobile readout’. It builds on the concept that lipid membranes can improve FET readout under high-ionic-strength conditions [9], while extending recent advances in transistor-based and integrated biosensing toward clinically relevant cardiovascular scenarios [6,7]. For acute coronary syndrome, where ‘time is myocardium’, a direct-serum, 15-min, low-instrument-dependence POCT route—with a reported cost below $2 per test—has clear practical significance [8].

**Figure 1.**
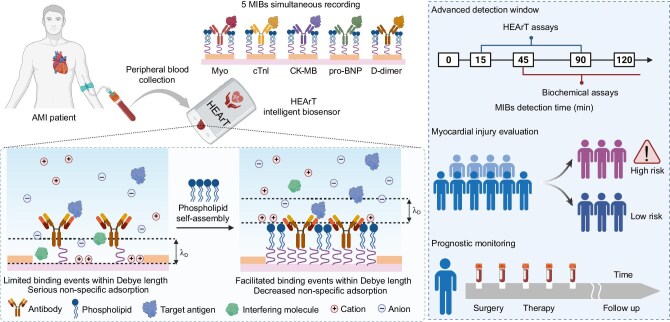
Schematic overview of the HEArT platform. A lipid monolayer-capped MPI-FET interface extends the effective electrical sensing range and forms a hydrated antifouling layer to reduce non-specific adsorption. The 5 biomarker array enables pg/mL-level detection, advances the AMI detection window by >30 min and supports rapid bedside screening, risk stratification and longitudinal post-percutaneous coronary intervention monitoring. MIBs, myocardial injury biomarkers. Reproduced from Fig. 1 of Ref. [8] with permission. Created by BioRender.

Several challenges remain. Current ultra-early AMI detection mainly relies on cTnI, Myo and CK-MB, and the cohort is limited [8]. Classification of some non-AMI cardiovascular subtypes remains modest, indicating that the five-marker panel does not yet fully capture CVD heterogeneity. HEArT is therefore best viewed as a highly translational prototype rather than a finalized clinical solution. Future integration of inflammatory markers, microRNAs (miRNAs) or vital-sign parameters, together with larger multi-center prospective validation, could substantially expand its impact in chest-pain centers, emergency triage and resource-limited settings.

Beyond its application in cardiovascular diagnostics, the broader methodological significance of the HEArT platform lies in demonstrating that a lipid-capped FET interface can overcome two long-standing barriers to electrical biosensing in physiological fluids: Debye screening caused by high ionic strength and non-specific protein fouling. By showing that a biomimetic phospholipid monolayer enables sensitive, specific and stable signal transduction directly in undiluted serum, this work provides a generalizable strategy for adapting FET-based biosensors to real-world biological matrices—potentially including blood, urine and interstitial fluid, pending matrix-specific validation. This interface-centric approach, rather than the specific biomarker panel, represents the key advance that may drive future development of point-of-care electronic assays across a wide range of diseases.
